# The increased risk of colorectal cancer in the women who underwent hysterectomy from the South Korean National Health Insurance Database

**DOI:** 10.1186/s12905-023-02642-3

**Published:** 2023-09-29

**Authors:** Jin -Sung Yuk, Seung-Woo Yang, Sang-Hee Yoon, Myoung Hwan Kim, Yong-Soo Seo, Yujin Lee, Jungbin Kim, Keunho Yang, Geumhee Gwak, Hyunjin Cho

**Affiliations:** 1 Department of Obstetrics and Gynecology, Sanggye Paik Hospital, School of Medicine, Inje University, Seoul, Republic of Korea; 2 Department of Surgery, Sanggye Paik Hospital, School of Medicine, Inje University, Seoul, Republic of Korea; 3 Department of Surgery, Sanggye Paik Hospital, School of Medicine, Inje University, Dongil-Ro, Nowon-Gu, Seoul, 1342 Republic of Korea

**Keywords:** Adnexal surgery, Colorectal cancer, Hysterectomy, Gastrointestinal cancer, Rectal cancer

## Abstract

**Background:**

Several population-based studies and observational studies have shown that oophorectomy is associated with an increased risk of colorectal cancer (CRC), and hormone replacement therapy has been associated with a reduction in the risk of colorectal cancer. This study was carried out to investigate whether hysterectomy, which may affect the levels of female hormones, is associated with a risk of cancer of the specific gastrointestinal tract.

**Methods:**

This population-based retrospective cohort study was conducted using insurance data provided by the Health Insurance Review and Assessment Service (HIRA) from January 1, 2007, to December 31, 2020. The hysterectomy group included 40- to 59-year-old women who underwent hysterectomy with uterine leiomyoma or uterine endometriosis from January 1, 2011, to December 31, 2014. The control group included women aged 40 to 59 years who visited medical institutions for medical examination from January 1, 2011 to December 31, 2014.

**Results:**

The hysterectomy and non-hysterectomhy groups comprised 66,204 and 89,768 subjects, respectively. The median ages in the non-hysterectomy group and hysterectomy group were 48 (range: 43–53) and 46 (range: 44–49) years, respectively. In the unadjusted results of the analysis, all colorectal cancer (CRC) increased in the hysterectomy alone group (HR 1.222, 95% confidence interval (CI) 1.016–1.47, *p* = 0.033), sigmoid colon cancer increased in the hysterectomy alone group (HR 1.71, 95% CI 1.073–2.724, *p* = 0.024), and rectal cancer increased in the hysterectomy with adnexal surgery group (HR 1.924, 95% CI 1.073–2.724, *p* = 0.002). The adjusted results showed that all CRC increased in the hysterectomy alone group (HR 1.406, 95% CI 1.057–1.871, *p* = 0.019), colon cancer increased in the hysterectomy alone group (HR 1.523, 95% CI 1.068–2.17, *p* = 0.02), and rectal cancer increased in the hysterectomy with adnexal surgery group (HR 1.933, 95% CI 1.131–3.302, *p* = 0.016). The all-cause mortality of GI cancer increased in the hysterectomy alone group (HR 3.495, 95% CI 1.347–9.07, *p* = 0.001).

**Conclusions:**

This study showed that the risk of all CRC increased in women who underwent hysterectomy compared with women who did not. In particular, the risk of rectal cancer was significantly higher in the women who underwent hysterectomy with adnexal surgery than in the controls. There was no association between hysterectomy and other GI cancers.

**Supplementary Information:**

The online version contains supplementary material available at 10.1186/s12905-023-02642-3.

## Introduction

Hysterectomy is one of the most frequent gynecological surgeries in the United States [1]. The majority of hysterectomies are performed because of benign conditions such as symptomatic uterine fibroids, endometriosis, or excessive uterine bleeding [2]. Although many women experience improved quality of life (QoL) after hysterectomy due to elimination of bothersome symptoms [3], hysterectomy is known to cause postoperative physiologic changes that are related to decreased QoL and poor health outcomes, including psychiatric morbidity, especially in younger women under 40 years of age [4, 5]. Additionally, hysterectomy may cause impaired ovarian function by damaging ovarian tissue or compromising the blood supply [6]. Many studies have shown that premenopausal women who had a hysterectomy with ovarian preservation have lower ovarian sex steroid levels and experience earlier menopause than women who do not have a hysterectomy [7, 8].

Adnexal surgery involves any of the organs that are on the sides of the uterus, such as fallopian tubes and ovaries. Bilateral oophorectomy is most often an elective procedure for the management of benign conditions such as chronic pelvic pain, ovarian cysts or tumors and risk reduction for hereditary ovarian cancer [9]. In contrast to with natural menopause, surgical menopause entails an abrupt withdrawal of estrogen, progesterone, and androgens, which are associated with more severe and prolonged menopausal symptoms, particularly when it is performed before the age of 45 years. Long-term consequences of oophorectomy include not only impaired cognitive and neurologic functions but also increased risks for cardio-metabolic disorder and bone resorption. There is a survival benefit to retention of the ovaries up to the age of 65 years in women at low risk for ovarian cancer [10]. Furthermore, a population-based study from the Swedish patient registry showed that oophorectomy increased the risk of colorectal cancer (CRC) [11]. A prospective cohort study from Denmark showed that oophorectomy was associated with an increased risk of CRC, with the highest rates being among women with bilateral oophorectomy [12].

In South Korea, CRC is the third most commonly diagnosed cancer in both sexes, following breast cancer and thyroid cancer in females and gastric cancer and lung cancer in males [13]. The incidence of CRC has recently increased dramatically in Korea. Globally, South Korea had the second highest CRC incidence in 2018, with an estimated 44.5 cases per 100,000 people per year [14]. The incidence of CRC is lower in women than in men at all ages, and estrogen exposure is known to have preventive effects, more so in women than in men [15].

The Women’s Health Initiative (WHI) clinical trial reported that relatively short-term use of estrogen plus progestin was associated with a decreased risk of CRC but that CRCs in women who took estrogen plus progestin were diagnosed at a more advanced stage than those in women who took placebo [16]. However, in the long-term treatment and follow-up of women for over 7 to 8 years, the hazard ratio (HR) patterns in the WHI clinical trial and observational study did not provide strong evidence of a clinically important CRC benefit with either estrogen alone or estrogen plus progestin [17]. Numerous epidemiological studies have shown that users of high-dose oral contraceptives and of menopausal hormone therapy (MHT) have a 20 to 40% lower incidence of CRC than nonusers [18–20]. Observational and experimental studies have revealed that exposure to oral contraceptives and MHT lowers the risk of CRC [21]. However, several cohort and case control studies have shown conflicting results regarding the risk of CRC and endogenous levels of sex steroids in postmenopausal women [22, 23].

As mentioned above, hysterectomy could affect the decrease in the endogenous levels of estrogen and progesterone and eventually increase the risk of CRC [7, 8]. Abundant previous studies have suggested that female sex hormones are associated with the risk of CRC [16, 18–21]. Therefore, we designed this study to evaluate the impact of hysterectomy or hysterectomy with adnexal surgery, which may cause early menopause or impairment of ovarian function, on the risk of gastrointestinal (GI) cancer using Korean health insurance data. The primary purpose of this study was to analyze the risk of CRC in women who underwent hysterectomy due to a nonmalignant disease, and the secondary purpose was to determine the risk of each GI cancer according to specific sites, such as the stomach, small bowel, and colon.

## Methods

Enrolment in the South Korean National Health Insurance Service (NHIS) is compulsory after birth registration and is lost on death. It is estimated that almost 99% of South Korean residents are covered by the National Health Service, as they gain or lose eligibility when they acquire or lose citizenship, such as through immigration [24]. The NHIS manages the medical record information (age, sex, prescribed drug name, diagnosis, type of medical insurance, operation name, hospitalization and outpatient care) of Koreans (approximately 51 million people) who are subscribed to the program. The Health Insurance Review Assessment (HIRA) is the review body for nationwide claims data from all hospitals in Korea. Hospitals in Korea are partially paid by patients according to the co-payment rate set by HIRA, and the remaining portions are paid by NHIS (National Health Insurance Service), the nationwide payer in Korea, after HIRA’s review for reimbursement adequacy. HIRA also provides data requested by researchers after review for study purposes. More details can be found on the official website: https://opendata.hira.or.kr/home.do (Korean only). This population-based retrospective cohort study was conducted using the database provided by HIRA from January 1^st^, 2007, to December 31^st^, 2020.

### Selection of participants

We used the International Classification of Diseases, 10th revision (ICD-10) and Korea Health Insurance Medical Care Exposes (2016 version: https://repository.hira.or.kr/handle/2019.oak/2119, 2019 version: https://repository.hira.or.kr/handle/2019.oak/2123) for the selection and analysis of subjects. From January 1^st^, 2011, to December 31^st^, 2014, women aged 40 to 59 who underwent hysterectomy with uterine myoma or endometrial disease were selected as the hysterectomy group. Adnexal surgery was performed by extirpation of the adnexal tumor (unilateral or bilateral oophorectomy, unilateral or bilateral salpingo-oophorectomy, unilateral or bilateral salpingectomy, unilateral or bilateral ovarian cystectomy, incision and drainage of the ovarian cyst, ovarian wedge resection, and adhesional adnexectomy, and hysterectomy and adnexal surgery were performed on the same day. The day of hysterectomy was determined as the inclusion day. The non-hysterectomy group included women aged 40–59 years who visited a medical institution for health checkups from January 1, 2011, to December 31, 2014, and females who had undergone hysterectomy before 2011 Jan 1^st^ were excluded in this study. The first visit to the health examination was designated the inclusion day.

We excluded subjects who had any of the following cancer diagnosis codes (any Cxx) or gastrointestinal disease codes within 180 days before enrollment: K25 (gastric ulcer), K26 (duodenal ulcer), K27 (peptic ulcer), K28 (gastrojejunal ulcer), K31.7 (polyp of stomach and duodenum), K50 (Crohn’s disease), K51 (ulcerative colitis), K63.5 (polyposis of colon), or D51.0 (pernicious anemia).

### Outcomes

Individuals were classified into the GI cancer group if they visited medical institutions more than 3 times with GI cancer diagnostic codes C15 (esophageal cancer), C16 (gastric cancer), C17 (cancer of small intestine), C18 (colon cancer; ascending, transverse, and descending colon), C19 (cancer of rectosigmoid junction; sigmoid cancer), or C20 (rectal cancer).

### Variables

The medical insurance type was defined as low socioeconomic status (SES) when it was medical protection; the residential area was defined as rural area if it was nonmetropolitan. The Charlson Comorbidity Index (CCI) was obtained using the diagnostic code from the selection date of the study to the year before [25]. In fact, all patients with co-morbidities such as hypertension, DM, dysthyroid, etc. were included in this study, which was adjusted with CCI. Parity was analyzed only for delivery in the entire cohort. We classified women who had a history of more than one adnexal surgery before hysterectomy as women with a history of adnexal surgery. We defined women who visited medical institutions more than once with a menopause-related diagnostic code before hysterectomy as menopause. The menopause-related diagnostic codes include N95.x (menopausal and other perimenopausal disorders), M80.0 (postmenopausal osteoporosis with pathological fracture), M81.0 (postmenopausal osteoporosis), and E28.3 (premature menopause), among others. Individuals who were prescribed their first MHT (menopausal hormone therapy) more than 180 days before the study were classified as having MHT before inclusion. Those who were prescribed their first MHT after inclusion were classified as having MHT after inclusion if the prescription date was 180 days or more after inclusion. MHT included tibolone, estradiol valerate, estradiol hemihydrate, dydrogesterone, norethisterone acetate, medroxyprogesterone acetate, drospirenone, and cyproterone, among others. Patients who visited medical institutions more than 3 times with gallbladder (GB) and biliary disease were defined as having gallbladder and biliary disease. The GB and biliary disease codes include K80 (Cholelithiasis), K81 (Cholecystitis), K82 (Other Diseases of gallbladder), K83 (Other Diseases of biliary tract), and K87 (Disorders of gallbladder, biliary tract and pancreas in diseases classification ed elsewhere), among others. The patients who visited medical institutions more than three times with uterine myoma (D25.x) or endometriosis (N80.x) were defined as having each of the relevant diseases. Death was defined as all cases of death during hospitalization.

### Statistics

SAS Enterprise Guide 7.15 (SAS Institute Inc. Cary, North Carolina, USA) and R 3.5.1 (R Foundation for Statistical Computing, Vienna, Austria) including survival package were used for statistical analysis. All statistical analyses were two-sided, and the results were defined as statistically significant if the *p* value was 0.05 or less. The analysis of categorical variables was carried out by Pearson’s chi-squared test or Fisher’s exact test, and t tests and Mann‒Whitney U tests were used for the analysis of continuous variables. We performed Cox regression analysis to correct the bias caused by confounding factors in the effect of hysterectomy on the risk of GI cancer. The first day for Cox analysis was set as the inclusion day of each group, and the last day was set as any GI cancer, the death date, or December 31^st^, 2020. We applied the listwise deletion method when the proportion of missing values for a statistical variable was less than 10% and the regression imputation method when the proportion of missing values was more than 10%. If there were more than 10% missing values, the SAS regression method (proc mi) was to be used, but there were no missing values. As this study uses the HIRA insurance data, it is linked to the national resident registration number. Therefore, there can be no missing values for age, place of residence and type of insurance. Even if there are exceptions, they are not included in the data provided by HIRA. Other variables are marked as ‘present’ only if the variable is applicable, and other variables are marked as ‘absent’ so that there are no missing values. For example, in the case of ‘adnexal surgery prior to enrolment’, only cases where the corresponding surgery code existed prior to the date of enrolment were marked as ‘present’. To confirm the robustness of our study, Cox regression analysis was performed on the risk of GI cancer in the laparotomic hysterectomy group (versus non-hysterectomy group).

### Ethics

This study have been performed in accordance with the Declaration of Helsinki. This study was approved and informed consent waived by the institutional review board (IRB) of Sanggye Paik Hospital (Approval number: SGPAIK 2021-12-005). In this study, the identifying variables of individuals were removed (deidentification). In addition, the analysis of this study was conducted only on closed servers provided by HIRA, and results data (e.g., tables, statistical values) can only be taken out of the server. Therefore, there is no harm to the participants who participated in this study because the individual cannot be specified. Additionally, raw data cannot be offered. Accordingly, this study does not require the provision of informed content to patients included in the data according to the Bioethics and Safety Act of South Korea. This study uses data provided by HIRA, but HIRA has no interest in this study.

## Results

**Fig. 1:**
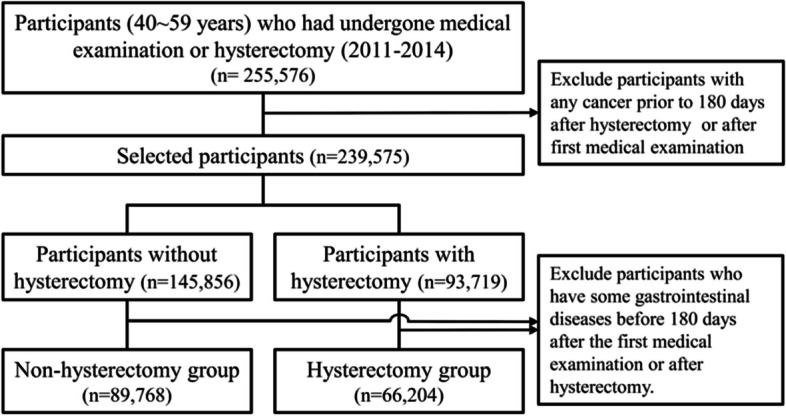
Study flowchart to select participants according to hysterectomy from the National Health Insurance Database, 2007–2020

Among a total of 155,972 subjects included, 66,204 and 89,768 subjects were classified into the hysterectomy and non-hysterectomy groups, respectively (Fig. 1). The baseline characteristics of participants with or without hysterectomy are shown in Table 1. The median age was 48 (range: 43–53) years in the non-hysterectomy group and 46 (range: 44–49) years in the hysterectomy group. The incidence of GI cancer among participants with/without hysterectomy according to specific sites of the GI tract is shown in Table 2. The number of subjects with any GI cancer was 533 (0.6%) in the non-hysterectomy group and 419 (0.6%) in the hysterectomy group. GI cancers were classified as esophageal cancer, stomach cancer, small bowel cancer, colon cancer (ascending, transverse, descending), sigmoid cancer, or rectal cancer. Esophageal cancer occurred in 8 subjects (0.009%) in the hysterectomy group and 9 subjects (0.009%) in the non-hysterectomy group (*p* = 0.975). There were 247 (0.3%) subjects with stomach cancer in the non-hysterectomy group and 158 (0.2%) in the hysterectomy group (*p* = 0.162). There were 15 (0.0%) subjects with small bowel cancer in the non-hysterectomy group and 10 (0.0%) in the hysterectomy group (*p* = 0.806). The number of subjects with colon cancer (ascending, transverse, descending) was 174 (0.2%) in the non-hysterectomy group and 162 (0.2%) in the hysterectomy group (*p* = 0.032). There were 35 (0.0%) subjects with sigmoid colon cancer in the non-hysterectomy group and 43 (0.1%) in the hysterectomy group (*p* = 0.023). There were 94 (0.1%) subjects with rectal cancer in the non-hysterectomy group and 94 (0.1%) in the hysterectomy group (*p* = 0.036). The number of subjects with total CRC was 265 (0.3%) in the non-hysterectomy group and 251 (0.4%) in the hysterectomy group (*p* = 0.004) (Table 2).

**Table 1:** Baseline characteristics of participants with/without hysterectomy from the Korea National Health Insurance Database, 2007-2020^a^

	Non-Hysterectomy, %	Hysterectomy, %	Total, %	*p*-value^†^
Number of participants	89,768	66,204	155,972	
Adnexal surgery during hysterectomy				
Hysterectomy		77.7	77.7	
Hysterectomy + adnexal surgery		22.3	22.3	
Median age (years)	48 [43–53]	46 [44–49]	47 [44–52]	< 0.001
Age at inclusion (years)				< 0.001
40 ~ 44	30.9	32.7	31.6	
45 ~ 49	24.2	43.7	32.5	
50 ~ 54	25.1	20.1	23.0	
55 ~ 59	19.8	3.6	12.9	
Year at inclusion				< 0.001
2011	21.3	29.1	24.6	
2012	22.6	25.8	24.0	
2013	27.3	23.2	25.5	
2014	28.9	21.9	25.9	
SES				< 0.001
Mid ~ high SES	96.8	97.7	97.2	
Low SES	3.2	2.3	2.8	
Region				< 0.001
Urban area	53.4	64.0	57.9	
Rural area	46.6	36.0	42.1	
CCI				< 0.001
0	74.3	76.9	75.4	
1	14.6	13.2	14	
≥ 2	11.1	9.9	10.6	
Parity in cohort				< 0.001
0	96	98.7	97.1	
1	2.5	0.9	1.9	
≥ 2	1.5	0.4	1.0	
Menopause before inclusion				< 0.001
Absent	79.4	91.8	84.6	
Present	20.6	8.2	15.4	
MHT before inclusion				< 0.001
Absent	96.1	98.6	97.1	
Present	3.9	1.4	2.9	
Adnexal surgery before inclusion				< 0.001
Absent	98.1	98.9	98.5	
Present	1.9	1.1	1.5	
Diseases of the gallbladder and biliary tract before inclusion				< 0.001
Absent	98.3	98.7	98.5	
Present	1.7	1.3	1.5	
Uterine leiomyoma				< 0.001
Absent	90.9	21.0	61.2	
Present	9.1	79.0	38.8	
Endometriosis				< 0.001
Absent	97.9	73.3	87.5	
Present	2.1	26.7	12.5	
MHT after inclusion				< 0.001
Absent	93.0	85.7	89.9	
Present	7.0	14.3	10.1	

*CCI* Charlson comorbidity index, *MHT* menopausal hormone therapy, *SES* socioeconomic status

^†^*p*-value less than 0.05 is statistically significant

^a^Data are expressed as percentage without number

**Table 2:** The incidence of gastrointestinal cancer in participants with/without hysterectomy from the Korea National Health Insurance Database, 2007-2020^a^

	Non-Hysterectomy (%)	Hysterectomy (%)	Total (%)	*p*-value^†^
Number of participants	89,768	66,204	155,972	
**Esophageal cancer**				0.975
Absent	89,760 (100)	66,198 (100)	155,958 (100)	
Present	8 (0.008)	6 (0.009)	14 (0.009)	
**Stomach cancer**				0.162
Absent	89,521 (99.7)	66,046 (99.8)	155,567 (99.7)	
Present	247 (0.275)	158 (0.238)	405 (0.260)	
**Small bowel cancer**				0.805
Absent	89,753 (100)	66,194 (100)	155,947 (100)	
Present	15 (0.017)	10 (0.015)	25 (0.016)	
**Colon cancer**				0.032
Absent	89,594 (99.8)	66,042 (99.8)	155,636 (99.8)	
Present	174 (0.194)	162 (0.225)	336 (0.215)	
**Sigmoid cancer**				0.023
Absent	89,733 (100)	66,161 (99.9)	155,894 (99.9)	
Present	35 (0.039)	43 (0.065)	78 (0.050)	
**Rectal cancer**				0.036
Absent	89,674 (99.9)	66,110 (99.9)	155,784 (99.9)	
Present	94 (0.105)	94 (0.142)	188 (0.121)	
**Colorectal cancer**				0.004
Absent	89,503 (99.7)	65,953 (99.6)	155,456 (99.7)	
Present	265 (0.295)	251 (0.379)	516 (0.331)	
**Gastrointestinal cancer**				0.327
Absent	89,235 (99.4)	65,785 (99.4)	155,020 (99.4)	
Present	533 (0.594)	419 (0.633)	952 (0.610)	

^†^*p*-value less than 0.05 is statistically significant

^a^Data are expressed as the number (%)

**Fig. 2:**
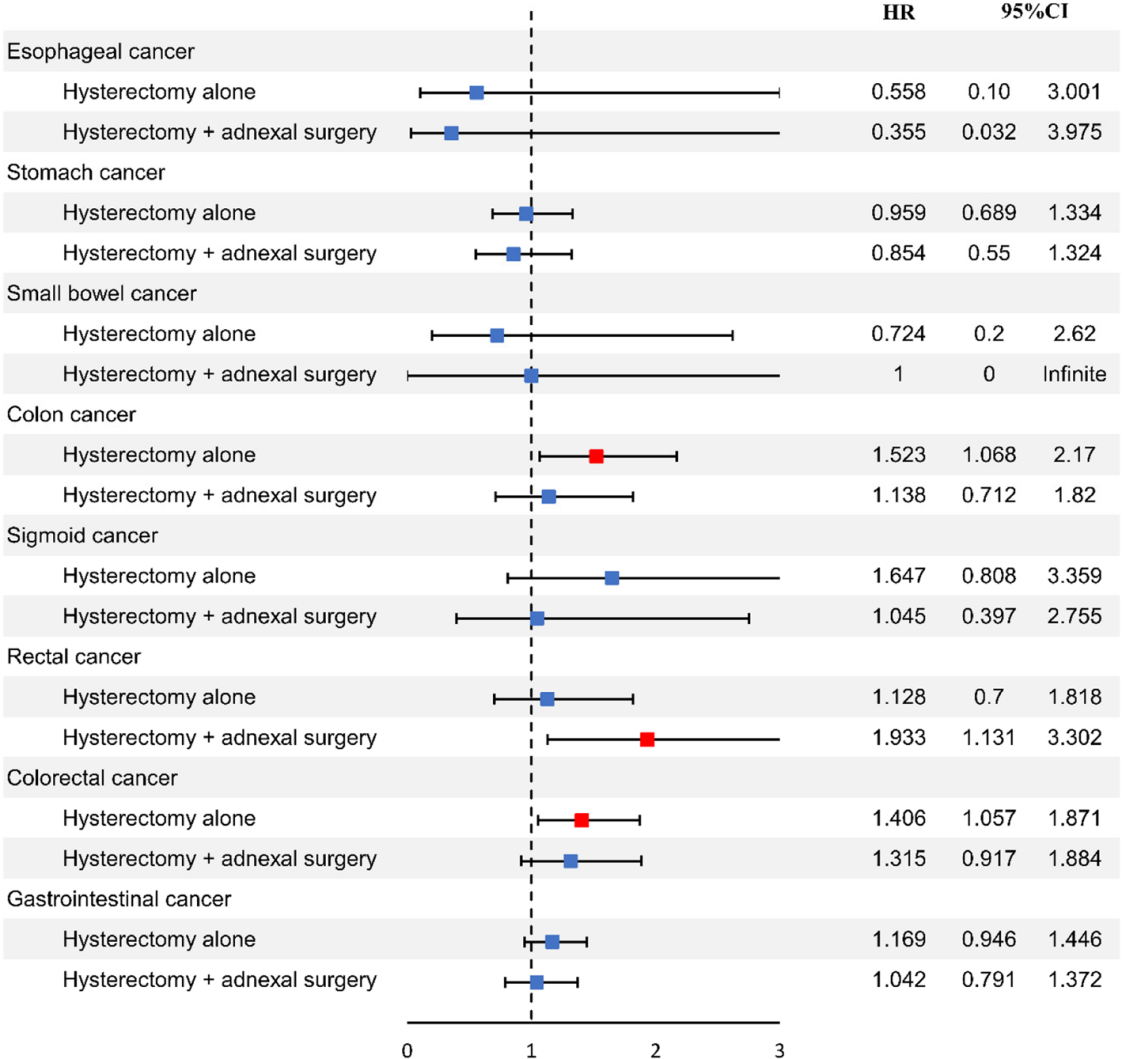
Association between hysterectomy with or without adnexal surgery and the risk of GI cancer

We analyzed the hazard ratios (HRs) of GI cancer with/without hysterectomy. The HRs were adjusted for hysterectomy, age, SES, region, CCI, parity, menopause before inclusion, MHT before inclusion, adnexal surgery before inclusion, diseases of the gallbladder and biliary tract before inclusion, uterine leiomyoma, and endometriosis. In the unadjusted results of the analysis, all colorectal cancer (CRC) increased in the hysterectomy alone group (HR 1.222, 95% confidence interval (CI) 1.016–1.47, *p* = 0.033), sigmoid colon cancer increased in the hysterectomy alone group (HR 1.71, 95% CI 1.073–2.724, *p* = 0.024), and rectal cancer increased in the hysterectomy with adnexal surgery group (HR 1.924, 95% CI 1.073–2.724, *p* = 0.002). Among the adjusted results of the analysis, the risk of esophageal cancer did not significantly change in the hysterectomy group (HR 0.558, 95% CI 0.104–3.001, *p* = 0.497) compared to the non-hysterectomy group. The risk of gastric cancer also did not significantly change in the hysterectomy group (HR 0.959, 95% CI 0.689–1.334, *p* = 0.805) compared to the non-hysterectomy group. All CRC increased in the hysterectomy-alone group (HR 1.406, 95% CI 1.057–1.871, *p* = 0.019), colon cancer increased in the hysterectomy-alone group (HR 1.523, 95% CI 1.068–2.17, *p* = 0.02), and rectal cancer increased in the hysterectomy with adnexal surgery group (HR 1.933, 95% CI 1.131–3.302, *p* = 0.016) (Table 3). The forest plot of the HRs associated with GI cancers in hysterectomy with or without adnexal surgery is shown in Fig. 2.

**Table 3:** Hazard ratios of gastrointestinal cancer in participants with/without hysterectomy from the Korea National Health Insurance Database, 2007–2020

	Unadjusted		Adjusted^a^	
	HR (95% CI)^a^	*P*-value	HR (95% CI)^a^	*p*-value^*^
**Esophageal cancer**				
Reference (no hysterectomy)	1 (reference)		1 (reference)	
Hysterectomy alone	1.031 (0.337–3.156)	0.957	0.558 (0.104–3.001)	0.497
Hysterectomy + adnexal surgery	0.726 (0.091–5.803)	0.763	0.355 (0.032–3.975)	0.401
**Stomach cancer**				
Reference (no hysterectomy)	1 (reference)		1 (reference)	
Hysterectomy alone	0.842 (0.679–1.045)	0.118	0.959 (0.689–1.334)	0.805
Hysterectomy + adnexal surgery	0.781 (0.543–1.124)	0.183	0.854 (0.55–1.324)	0.48
**Small bowel cancer**				
Reference (no hysterectomy)	1 (reference)		1 (reference)	
Hysterectomy alone	1.069 (0.479–2.382)	0.871	0.724 (0.2–2.62)	0.622
Hysterectomy + adnexal surgery	1 (0-Infinite)	0.984	1 (0-Infinite)	0.99
**Colon cancer**				
Reference (no hysterectomy)	1 (reference)		1 (reference)	
Hysterectomy alone	1.249 (0.996–1.566)	0.055	1.523 (1.068–2.17)	0.02
Hysterectomy + adnexal surgery	0.999 (0.678–1.471)	0.994	1.138 (0.712–1.82)	0.589
**Sigmoid cancer**				
Reference (no hysterectomy)	1 (reference)		1 (reference)	
Hysterectomy alone	1.71 (1.073–2.724)	0.024	1.647 (0.808–3.359)	0.17
Hysterectomy + adnexal surgery	1.168 (0.519–2.63)	0.708	1.045 (0.397–2.755)	0.929
**Rectal cancer**				
Reference (no hysterectomy)	1 (reference)		1 (reference)	
Hysterectomy alone	1.113 (0.808–1.531)	0.513	1.128 (0.7–1.818)	0.62
Hysterectomy + adnexal surgery	1.924 (1.282–2.888)	0.002	1.933 (1.131–3.302)	0.016
**Colorectal cancer**				
Reference (no hysterectomy)	1 (reference)		1 (reference)	
Hysterectomy alone	1.222 (1.016–1.47)	0.033	1.406 (1.057–1.871)	0.019
Hysterectomy + adnexal surgery	1.206 (0.902–1.613)	0.206	1.315 (0.917–1.884)	0.136
**Gastrointestinal cancer**				
Reference (no hysterectomy)	1 (reference)		1 (reference)	
Hysterectomy alone	1.029 (0.897–1.181)	0.68	1.169 (0.946–1.446)	0.149
Hysterectomy + adnexal surgery	0.963 (0.768–1.206)	0.741	1.042 (0.791–1.372)	0.772

*CCI* Charlson comorbidity index, *CI* confidence interval, *HR* hazard ratio, *MHT* menopausal hormone therapy, *SES* socioeconomic status

^*^p-value less than 0.05 is statistically significant

^a^HRs were adjusted for hysterectomy, age, SES, regrion, CCI, parity, menopause before inclusion, MHT before inclusion, adnexal surgery before inclusion, diseases of the gallbladder and biliary tract before inclusion, uterine leiomyoma, endometriosis

The HRs of all-cause mortality among participants with GI cancer are shown in Table 4. Among the adjusted results, the mortality of GI cancer increased in the hysterectomy alone group (HR 3.495, 95% CI 1.347–9.07, *p* = 0.001).

**Table 4:** Hazard ratios of all-cause mortality in participants with Gastrointestinal cancer from the Korea National Health Insurance Database, 2007–2020

	Adjusted^a^	
	HR (95% CI)^a^	*p*-value^*^
**Colon cancer**		
Reference (no hysterectomy)	1 (reference)	
Hysterectomy alone	2.405 (0.385–15.018)	0.348
Hysterectomy + adnexal surgery	1 (0-Infinite)	0.993
**Colorectal cancer**		
Reference (no hysterectomy)	1 (reference)	
Hysterectomy alone	3.369 (0.641–17.719)	0.152
Hysterectomy + adnexal surgery	1 (0-Infinite)	0.991
**Gastrointestinal cancer**		
Reference (no hysterectomy)	1 (reference)	
Hysterectomy alone	3.495 (1.347–9.07)	0.001
Hysterectomy + adnexal surgery	1.913 (0.535–6.844)	0.319

*CCI* Charlson comorbidity index, *CI* confidence interval, *HR* hazard ratio, *MHT* menopausal hormone therapy, *SES* socioeconomic status

^*^p-value less than 0.05 is statistically significant

^a^HRs were adjusted for hysterectomy, age, SES, regrion, CCI, parity, menopause before inclusion, MHT before inclusion, adnexal surgery before inclusion, diseases of the gallbladder and biliary tract before inclusion, uterine leiomyoma, endometriosis

## Discussion

This study showed that the risk of all CRC was higher in women who underwent hysterectomy than in women who did not. In particular, the risk of rectal cancer was significantly higher in the women who underwent hysterectomy with adnexal surgery than in the non-hysterectomy group. This result is consistent with a systematic review suggesting that oophorectomy or hysterectomy increases the subsequent risk of CRC and should not be recommended for women who are not at high risk except for those with susceptibility genes for ovarian cancer [26]. This meta-analysis included 19 trials with 37,958 participants from Sweden, the United States and Finland, which were divided into three subgroups: the first group consisted of 10 trials comparing hysterectomy with no surgery, the second group consisted of 4 trials on the risk of CRC in oophorectomy compared with the general population, and the third group consisted of 5 trials on the risk of CRC in hysterectomy with bilateral salpingo-oophorectomy compared with simple hysterectomy. After sensitivity analyses and checking for publication bias in all the trials, they concluded that the risk of CRC was increased in the hysterectomy, oophorectomy, and hysterectomy with bilateral salpingo-oophorectomy groups compared with their respective controls [11]. However, Boggs et al. reported that there were no significant increases in the risk of CRC in women with oophorectomy at a younger age less than 40 and short duration of menopausal hormone use less than 2 years [27]. Furthermore, Luoto et al. reported that hysterectomy is not associated with any substantial protective or promoting effect on cancers in general [28].

Furthermore, the most recent study from the Danish Nurse Cohort showed that Bilateral oophorectomy was associated with a increase in CRC incidence, with a CRC-adjusted rate ratio (aRR) of 1.79 (95% CI 1.33–2.42). The effect estimate after unilateral oophorectomy was also higher, with an aRR of 1.25 (95% CI 0.86–1.82), but this was not statistically significant. The Danish Nurse Cohort Study has many strengths in terms of objective case ascertainment (unilateral/bilateral oophorectomy) and outcome incidence assessment based on the Danish registries, high quality data providing well characterized baseline information (such as BMI, parity, age at menarche and oral contraceptive use), homogeneity in ethnicity (98% Caucasian) and low risk of selection bias as all Danish nurses who were members of the Danish Nursing Organization were invited to participate in this cohort [12]. Our study is a retrospective case–control study comparing the incidence of GI cancer in patients undergoing uterine and adnexal surgery with a control group of healthy women without uterine and adnexal surgery. In contrast, the Danish Nurse Cohort Study compared the incidence of CRC in a single cohort of women with and without uterine and adnexal surgery. The Danish Nurse Cohort Study may be more concrete in the sense that it is less likely to be influenced by confounding variables and therefore more likely to be a true predictor of the outcomes.

Many studies have reported that hysterectomy itself, regardless of adnexal surgery, causes impairment mostly of ovarian function, which may decrease the protective effect of endogenous estrogen against CRC [6–8]. This prior evidence supports our observation that the risk of CRC, particularly rectal cancer, was higher in women with a hysterectomy than in those without a hysterectomy.

The risk factors for CRC include red and processed meat, body fat composition, alcohol consumption, smoking, and male sex [29, 30]. Sex disparities in not only the risk of CRC but also the prognosis of metastatic CRC have led to controversies regarding the role of sex hormones [31]. Estrogen, especially estradiol, is known to affect the onset of CRC in various laboratory and clinical studies [32, 33]. The morbidity and mortality of CRC are higher in men than in women because estrogen is associated with various growth factors affecting cell proliferation and microscopic changes in the cell immune response by binding estrogen receptor beta (ERβ) in the colon [34]. Furthermore, both estrogen and progestin reduce the serum levels of fasting blood sugar and insulin, which explains why hyperglycemia and hyperinsulinemia increase the risk of CRC [35, 36].

On the other hand, one study showed that colonic tumorigenesis is promoted by male hormones rather than decreased by female hormones [37]. Taken together, these findings suggest that estrogen in women and testosterone in men underlie a notably large sex disparity in the risk of CRC. There is a growing body of evidence supporting the notion that both female and male sex hormones can influence the onset of CRC. However, the ovaries of postmenopausal women do not produce female sex hormones but still produce substantial amounts of androgens [38, 39]. The postmenopausal ovary remains a critical source of androgen throughout the lifespan of older women within the 50–89 year age range [40]. The persistent secretion of androgens together with decreased levels of sex hormone binding globulin (SHBG) in the circulation during menopause provides an increased biological availability of androgens [41]. Long-term androgen deprivation therapy for prostate cancer is associated with an increased risk of CRC [42]. While the physiological effects of androgens are maintained in postmenopausal women with an intact uterus and ovaries, these protective effects of androgens cannot be maintained in women who have undergone surgical removal of the uterus and ovaries. These protective effects of androgen support the results of our study that hysterectomy with or without adnexal surgery is associated with an increased risk of CRC regardless of the use of MHT in postmenopausal women. After menopause, the absolute amount of androgen decreases, but it is assumed to play a role in the risk reduction of CRC because it is secreted continuously until the age of 90 in postmenopausal women with an intact uterus and ovaries. However, in our study, there was no difference in the risk of CRC when adnexal surgery was performed. Therefore, further study is needed to identify more clearly the physiological role of the ovaries after menopause.

Most previous studies with MHT have reported that the decreased risk of CRC is related to the use of estrogen and/or progestin in women without hysterectomy but not to estrogen alone in women with hysterectomy [18–21]. A logical explanation for the role of progestins in the protection of CRC is that progestins can increase the estrogenic effects of conjugated estrogens, making them more biologically active in the colon in women without hysterectomy than estrogen alone in women with hysterectomy. Meijer et al. reported that CRC prevention by progestin medroxyprogesterone acetate (MPA) is critically dependent on postmenopausal status. They found that MPA reduces tumorigenesis in postmenopausal mice but not in fertile mice [43]. Zhang et al. demonstrated that progesterone suppressed proliferation in CRC cells by arresting the cell cycle and inducing apoptosis. Moreover, progesterone-induced inhibition of CRC progression was regulated by GADD45α/JNK/c-Jun signaling [44].

Since the discovery of two types of estrogen receptor, namely, ERα [45, 46] and ERβ [47, 48], it has become clear that estrogen has diverse and complex effects in various tissues. ERα and ERβ have different biological functions in both nuclear and extranuclear signaling. ERα promotes proliferative signaling through differential expression of pro- and antiapoptotic proteins [49]. However, ERβ plays a role as a dominant regulator reducing ERα-mediated gene expression, which results in a consequent negative effect on cell proliferation [50]. On the other hand, ERβ has antiproliferative effects in the absence of ERα by activating proapoptotic signaling [51]. Numerous studies have suggested that ERβ is essential for the maintenance of cellular homeostasis and for driving cellular differentiation in the colon [52]. In vitro and in vivo studies showing that ERβ is more highly expressed in colonic epithelial cells than ERα strongly suggest a functional implication for ERβ in mediating the effects of estrogens on colonic epithelial cancer cells [53] and in the protective effects against CRC [54].

Additionally, estrogen is emerging as an important regulator of bile acid (BA) production and, through critical hepatic feedback mechanisms, serum cholesterol levels [55]. BAs could act as tumor promoters in the colon. Approximately 90 ~ 95% of the BA pool is reabsorbed in the ileum, transported back to the liver via the hepatic portal vein and ready for new enterohepatic circulation. Approximately 5 ~ 10% of the total BA pool escapes from ileum reabsorption and flows to the colon, where some of it is deconjugated by bacterial bile salt hydrolases to become free BA and converted to secondary BAs. Less than 10% of these BAs are lost via feces [56]. Bacterial conversion of BAs in the colon has a significant impact on their tumorigenic activity through its effects on colonic microbial metabolism [57]. While hydrophilic, less cytotoxic BAs play a protective role in GI and liver cells, hydrophobic BAs can be cytotoxic and can generate oxidative stress and DNA damage, which affects the development of cancer in various digestive and extra-digestive organs by affecting epigenetic factors and changes in intestinal microbes [58]. Women have fewer BAs excreted in the stool and less cholesterol through bile production than men [59]. Therefore, a decrease in estrogen in women who undergo hysterectomy may contribute to the risk of developing CRC by increasing the proportion of BAs in the intestine.

Finally, interactions between the immune system and the gut microbiome are fundamental to the maintenance of immune homeostasis in the human organism. Many studies show that gut microbiota dysbiosis may cause bowel inflammation, irritable bowel syndrome and colorectal cancer, as well as diabetes, mastitis and polycystic ovarian syndrome [60]. Endometriosis, one of the most common gynecological conditions for which hysterectomy is considered, is thought to be closely related to immune disorders, sharing features with autoimmune diseases such as reduced apoptosis, elevated cytokines and abnormal cell-mediated pathways [61]. In a recent study, researchers reviewed a number of studies that attempted to identify changes in the core gut microbiome of patients with endometriosis at different clinical stages of the disease. The researchers suggest that this could open the way to the use of probiotic treatment prior to surgical intervention, which many patients prefer to avoid [62]. In addition, Wang et al., using high-throughput 16S rRNA gene sequencing, reported that trans-abdominal hysterectomy alters the gut microbiota by reducing estrogen levels in the body, thereby reducing the diversity and abundance of the gut microbiota [63]. If we could not only detect the dysbiosis in endometriosis at an earlier stage of the disease, but might also be able to avoid hysterectomy, we could introduce new management strategies to prevent the progression of the disease and reduce the risk of fatal consequences such as CRC from the inflammatory process of the bowel.One of the notable findings of our study is that the risk of rectal cancer had the highest HR of 1.933 in women who underwent hysterectomy and adnexal surgery among all CRC patients. However, we cannot explain this finding because we had difficulty finding previous studies with differential analysis for each colon cancer and rectal cancer. Further studies must be performed to determine the real impact of hysterectomy with adnexal surgery on the risk of rectal cancer in the future. The overall risk of GI cancer was not different between the groups with or without hysterectomy, but the risk of CRC increased in women who had hysterectomy. When we divided all GI cancers into upper GI (UGI) and lower GI (LGI) cancers, the risk of UGI cancer did not increase, but the risk of LGI cancer increased in women who underwent hysterectomy. These results are consistent with many epidemiologic studies showing that carcinogenesis of the UGI tract, especially esophageal and gastric cancer, is highly dependent on several medical conditions, such as gastroesophageal reflux disease (GERD), Barrett’s esophagus, use of acid-suppressive medication or nonsteroidal anti-inflammatory drugs, and major environmental factors, such as tobacco use, diet, alcohol drinking, and *Helicobacter pylori* infection [64]. However, unlike the risk factors for UGI cancer, the risk factors for LGI cancer include female hormones in addition to environmental factors.

The strength of our study is that we analyzed the risk of cancer according to each portion of the GI track from the stomach to rectum in women who had a hysterectomy with/without adnexal surgery using big data from the national health care system of South Korea. Our study has some limitations. First, although adjustments were made for the numerous factors related to the risk of CRC, we must be careful in the interpretation of our results because this study has the inborn limitation of retrospective cohort studies. Second, our study is a one-sided sex analysis, which means that this study could ignore the complex effects sex-related hormones on the risk of CRC.

## Conclusion

This study showed that the risk of all CRC was higher in women who underwent hysterectomy than in women who did not. In particular, the risk of rectal cancer was significantly higher in the women who underwent hysterectomy with adnexal surgery than in the controls.

### Supplementary Information

**Additional file 1: Supplementary Table 1.** Case/person-years of gastrointestinal cancer in participants with or without hysterectomy. **Supplementary Table 2.** Hazard ratios of gastrointestinal cancer in participants with/without hysterectomy according to age group.

## Data Availability

All data generated or analysed during this study are included in this published article and its Supplementary information files. The datasets generated and/or analysed during the current study are not publicly available. This is because the dataset for this study is only available on the NHIS servers for one year after the dataset was generated. Therefore, the data of the series will not be available for sharing by bona fide researchers or for further statistical analysis in the future. However, upon reasonable request, the corresponding author will consider a response to explain the details of the data.

## Abbreviations

Bas: Bile acids
BC: Breast cancer
BMI: Body mass index
BPA: Bisphenol A
BWHS: Black women’s health study
CCI: Charlson comorbidity index
CI: Confidence interval
CIS: Carcinoma in situ
CRC: Colorectal cancer
DM: Diabetes mellitus
E2: 17β-Estradiol
ER: Estrogen receptor
P4: Progesterone
GERD: Gastroesophageal reflux disease
GI: Gastrointestinal
HIRA: Health insurance review and assessment service
HR: Hazard ratio
HTN: Hypertension
ICD-10: International classification of diseases, 10th revision
IRB: Institutional review board
LGI: Lower gastrointestinal
MHT: Menopausal hormone therapy
MPA: Medroxyprogesterone acetate
NHIS: National health insurance service
QoL: Quality of life
RCT: Randomized clinical trial
SES: Socioeconomic status
SHBG: Sex hormone binding globulin
UGI: Upper gastrointestinal
WHI: Women’s Health Initiative
