# Supplementary material for: The increased risk of colorectal cancer in the women who underwent hysterectomy from the South Korean National Health Insurance Database
Source: BMC Womens Health. 2023 Sep 29;23:519. doi: 10.1186/s12905-023-02642-3 (PMC10542264; doi:10.1186/s12905-023-02642-3)
Supplement: Supplementary file 1 — Additional file 1: Supplementary Table 1. Case/person-years of gastrointestinal cancer in participants with or without hysterectomy. Supplementary Table 2. Hazard ratios of gastrointestinal cancer in participants with/without hysterectomy according to age group. [file 12905_2023_2642_MOESM1_ESM.docx]

| Supplementary Table 1. Case/person-years of gastrointestinal cancer in participants with or without hysterectomy. | | | | |
| --- | --- | --- | --- | --- |
|  | Non-Hysterectomy | Hysterectomy | Hysterectomy + adnexal surgery |  |
| Total | 533/698,467 (76) | 331/417,598 (79) | 88/119,011 (74) |  |
| Age at inclusion (years) |  |  |  |  |
| 40~44 | 106/214,611 (49) | 83/142,787 (58) | 21/33,266 (63) |  |
| 45~49 | 124/169,503 (73) | 159/185,596 (86) | 37/48,458 (76) |  |
| 50~54 | 141/176,235 (80) | 77/77,663 (99) | 25/29,550 (85) |  |
| 50~55 | 162/138,117 (117) | 12/11,552 (104) | 5/7,736 (65) |  |
| SES |  |  |  |  |
| Mid~high SES | 513/675,870 (76) | 322/408,074 (79) | 88/116,422 (76) |  |
| Low SES | 20/22,597 (89) | 9/9,524 (94) | /2,589 (0) |  |
| Region |  |  |  |  |
| Urban area | 284/369,960 (77) | 206/263,147 (78) | 60/80,547 (74) |  |
| Rural area | 249/328,506 (76) | 125/154,451 (81) | 28/38,464 (73) |  |
| CCI |  |  |  |  |
| 0 | 383/518,376 (74) | 255/322,378 (79) | 61/90,231 (68) |  |
| 1 | 76/102,247 (74) | 39/54,216 (72) | 17/16,886 (101) |  |
| ≥2 | 74/77,843 (95) | 37/41,004 (90) | 10/11,895 (84) |  |
| Parity in cohort |  |  |  |  |
| 0 | 524/672,253 (78) | 329/412,154 (80) | 88/117,901 (75) |  |
| 1 | 6/16,715 (36) | 1/3,847 (26) | /811 (0) |  |
| ≥2 | 3/9,498 (32) | 1/1,598 (63) | /298 (0) |  |
| Menopause before inclusion |  |  |  |  |
| Absent | 406/553,559 (73) | 314/385,959 (81) | 80/106,408 (75) |  |
| Present | 127/144,907 (88) | 17/31,639 (54) | 8/12,603 (63) |  |
| MHT before inclusion |  |  |  |  |
| Absent | 513/671,553 (76) | 327/412,225 (79) | 87/117,068 (74) |  |
| Present | 20/26,914 (74) | 4/5,373 (74) | 1/1,943 (51) |  |
| Adnexal surgery before inclusion |  |  |  |  |
| Absent | 522/685,927 (76) | 328/414,075 (79) | 87/116,940 (74) |  |
| Present | 11/12,540 (88) | 3/3,523 (85) | 1/2,071 (48) |  |
| Diseases of the gallbladder and biliary tract before inclusion |  |  |  |  |
| Absent | 521/687,052 (76) | 327/412,336 (79) | 87/117,309 (74) |  |
| Present | 12/11,415 (105) | 4/5,262 (76) | 1/1,702 (59) |  |
| Uterine leiomyoma |  |  |  |  |
| Absent | 492/637,380 (77) | 66/85,760 (77) | 21/27,746 (76) |  |
| Present | 41/61,087 (67) | 265/331,839 (80) | 67/91,265 (73) |  |
| Endometriosis |  |  |  |  |
| Absent | 523/684,792 (76) | 254/312,832 (81) | 65/82,683 (79) |  |
| Present | 10/13,675 (73) | 77/104,766 (73) | 23/36,328 (63) |  |
| MHT after inclusion |  |  |  |  |
| Absent | 501/647,402 (77) | 306/363,692 (84) | 72/95,178 (76) |  |
| Present | 32/51,065 (63) | 25/53,906 (46) | 16/23,833 (67) |  |
|  |  |  |  |  |
| CCI, Charlson comorbidity index; MHT, menopausal hormone therapy; SES, socioeconomic status | | | | |
| Data are expressed as the case/person-years ( case/ 100,000 person-years). | | | | |

| Supplementary table 2. Hazard ratios of gastrointestinal cancer in participants with/without hysterectomy according to age group. | | | | | |
| --- | --- | --- | --- | --- | --- |
|  | 40~49 years ^a^ | | | 50~59 years ^a^ | |
|  | HR (95% CI) ^a^ | P-value | HR (95% CI) ^a^ | | P-value |
| Colon cancer |  |  |  | |  |
| Reference (no hysterectomy) | 1 (reference) |  | 1 (reference) | |  |
| Hysterectomy | 2.063 (1.309-3.251) | 0.002 | 0.995 (0.558-1.776) | | 0.987 |
| Hysterectomy + adnexal surgery | 1.907 (1.058-3.436) | 0.032 | 0.542 (0.236-1.245) | | 0.149 |
| Gastrointestinal cancer |  |  |  | |  |
| Reference (no hysterectomy) | 1 (reference) |  | 1 (reference) | |  |
| Hysterectomy | 1.194 (0.917-1.555) | 0.188 | 1.148 (0.804-1.641) | | 0.447 |
| Hysterectomy + adnexal surgery | 1.16 (0.818-1.646) | 0.405 | 0.915 (0.581-1.439) | | 0.699 |
|  |  |  |  | |  |
| CCI, Charlson comorbidity index; CI, confidence interval; HR, hazard ratio; MHT, menopausal hormone therapy;POP, pelvic organ prolapse; Gastrointestinal cancer, stress urinary incontinence; SES, socioeconomic status | | | | | |
| ^a^ HRs were adjusted for hysterectomy, age, SES, regrion, CCI, parity, menopause before inclusion, MHT before inclusion, adnexal surgery before inclusion, diseases of the gallbladder and biliary tract before inclusion, uterine leiomyoma, endometriosis. | | | | | |
